# Adverse events associated with chimeric antigen receptor T-cell therapy in ophthalmology: a narrative review

**DOI:** 10.1097/MS9.0000000000002188

**Published:** 2024-05-20

**Authors:** Sara Sarwar, Unood Riaz, Abraish Ali, Sejal Jain Kailash

**Affiliations:** a Department of Medicine, Dow University of Health Sciences, Karachi, Pakistan; b Department of medicine, Vinnytsia National Medical University, Vinnytsia, Ukraine

**Keywords:** adverse events, CAR-T cell, ophthalmic complications

## Abstract

Chimeric antigen receptors are synthetically produced receptors engineered to engage with target cells with high specificity. These cells are created by inserting an artificial T-cell receptor into an immunoglobulin’s antigen-binding region, allowing the cells to combine and target specific antigens. The use of chimeric antigen receptor (CAR) T-cell therapy has been a remarkable achievement in the field of immunotherapy, particularly in the treatment of ophthalmic tumors like retinoblastoma and uveal melanoma. However, there are some documented side effects, such as cytokine release syndrome (CRS) and immunological effector cell-associated neurotoxicity syndrome (ICANS). Additionally, ocular side effects such as blurred vision, vision impairment, and intraocular infections are also concerning and require further evaluation. This review highlights the advances made in chimeric antigen receptor (CAR) immunotherapy, including its structure and manufacture, as well as relevant clinical discoveries and associated adverse effects. By identifying the gaps in current research, this analysis provides insights into potential strategies and solutions for addressing some of the most severe side effects.

## Introduction

Highlights

- This study discusses the approval of FDA of the chimera antigen receptor T-cell therapy and its application in hematologic malignancies, lymphomas, autoimmune diseases and solid tumors. The immunotherapy involves targeted T-cell-mediated anti-tumor potency, and cytokine activity.
- Initial results with these treatments for intraocular malignancies such retinoblastomas and uveal melanomas were encouraging, which encouraged the use of genetically modified T cells more widely.
- Despite its effectiveness, CAR-T-cell therapy has a number of side effects, including neurotoxicity (ICAN mediated) and cytokine release syndrome (CRS), both of which have received substantial media coverage. However, nothing is known about the side effects of CAR-T-cell therapy on ocular illnesses. These include blurred vision and vision loss, optic neuropathies, and prolonged cytopenia eventually leading to an immunocompromised state.
- Data comprising of various case reports have opened up a new set of ophthalmic complications that were not identified earlier indicating the need for identification of these adverse effects along with routine ophthalmic follow-up for intraocular relapse as well as suitable guidelines for the usage.

Chimeric antigen receptor (CAR) T-cell therapy is an emerging immunotherapy that has played a significant role in the treatment of various oncological and hematological conditions since its first model, called the first-generation CAR-T cells^1^. The U.S. Food and Drug Administration (FDA) first approved Yescarta (axicabtagene ciloleucel), to treat large B-cell lymphoma and non-Hodgkin lymphoma (NHL), and since then, it has shown remarkable success in treating these hematological malignancies^2^. There have been massive advances in the field of CAR-T-cell therapy, as researchers are successfully engineering CAR-T cells with increased specificity to several tumors as well as extending to treat autoimmune diseases and viral infections^3,4^. Pre-clinical trials, and clinical studies indicate that CAR-T-cell therapy could be utilized for pediatric gliomas^2,5^ medulloblastoma, ependymoma^6^, rhabdomyosarcoma^7^, and various other sarcomas^8,9^. Combination therapies, like CAR-T-cell therapy with hematopoietic stem cell transplant (HSCT) and chemotherapy, are also being explored^10^.

CAR-T therapy has shown promising results in treating hematological malignancies. However, its clinical application presents several challenges, including cytokine release syndrome (CRS)^11^, limited efficacy in solid tumors, and high recurrence rates^12^. Therefore, it is crucial for ophthalmologists and oncologists to understand the potential ocular toxicities associated with this therapy. Recognizing the appropriate time to refer patients for an ophthalmic examination is essential in effectively managing these potential side effects^13^.

## Search strategy

Data on adverse events from this therapy continues to evolve. To explore the ocular consequences of CAR-T-cell therapy, we conducted a thorough search of PubMed’s database from inception to February 2023, utilizing medical subject headings and free-text terms for “receptors, chimeric antigen”, “eye”, “optic”, “ocular”, “ophthalmic” and “adverse effects” separated by Boolean operators AND and OR. To ensure comprehensive results, we also used synonyms and limited our search to English language articles. Studies were included if they met the following inclusion criteria: 1. Randomized controlled trials, 2. Clinical trials, 3. Case reports and series, 4. Reviews, 5. Patients undergoing CAR-T-cell therapy or those who received it in the past year and reported ophthalmic complications.

## Chimeric antigen receptor T-cell receptor therapy: structure and production

Many tumor-associated antigens (TAA) represent specific proteins used to support tumor genesis, transformation, and creation of a malignant phenotype. While some efforts have focused on expressing naturally occurring, tumor-specific TCRs in T cells, many groups have instead developed artificial receptors that are engineered to bind specifically to TAAs^14,15^, called CARs.

CAR-T-cell therapy is targeted immunotherapy that involves using T lymphocytes, which are either collected from the patient or a donor and genetically modified to attack specific tumor-specific antigens^16^. The process involves five key stages, which include removing red blood cells through leukapheresis, cell enrichment using paramagnetic beads and either a positive or negative selection approach, transducing T cells with CAR genes that target specific antigens like CD19 or B-cell maturation antigen (BCMA) using viral or non-viral approaches, growing and expanding the CAR-T cells using a medium containing recombinant interleukin 2 (IL-2) and other cytokines, and finally cryopreserving and storing the expanded CAR-T cells in liquid nitrogen before injection.

CARs are designed to identify specific antigens. While some CARs rely on an antigen-binding motif taken from antibodies, others use targeting moieties from receptors or ligand counterparts like heregulin or IL13. It is worth noting that CARs don’t need to match a patient’s HLA and can recognize cancers even when HLA expression is decreased^17^. These receptors have been evolving for over three decades, advancing both immunology and technology^1^. The latest CARs have even more antitumoral potency, cytokine activity, and stimulatory ligands and enzymes that can degrade the extracellular matrix in solid tumors^18^.

## Efficacy of CAR-T-cell therapy in ocular diseases

### Retinoblastoma

Retinoblastoma is the most frequent pediatric intraocular tumor. It is a rare malignancy that typically results due to a biallelic mutation of the retinoblastoma gene (RB1). The incidence of retinoblastoma is 1 in 16 000–18 000 live births worldwide^19^. Enucleation has traditionally been the mainstay of therapy, major centers have, however, reduced their enucleation rates over the past three decades in favor of globe-salvaging methods^20^. Researchers are currently exploring CAR-T-cell therapy as a potential alternative to improve treatment outcomes.

GD2 ganglioside and CD171 have been discovered as potential targets for CAR-T-cell therapy for retinoblastoma. Andersch and his colleagues demonstrated the effectiveness of CAR-T-cell therapy by using an in-vitro model. It was discovered that both CD171, and GD2 CAR-T cells displayed dose-dependent cytotoxicity of retinoblastoma cells, independently, and that sequentially targeting CD171 before GD2 enhanced tumor cell killing. After determining that the in-vitro model is generally successful, the Andersch group intends to proceed with in-vivo testing^21^.

Sujjitjoon and colleagues also examined GD2 CAR-T cells in an in-vitro retinoblastoma model. When they were co-cultured with the retinoblastoma cell line, ~100% of the cells died within 3 days. In contrast, the CD19 CAR-T cells were unable to effectively kill retinoblastoma cells^22^.

In 2020, Wang and colleagues used an in-vivo mouse model to show the efficacy of GD2 CAR-T cells. They altered their CAR-T-cell construct to release interleukin 15 (IL-15), which aids in T-cell proliferation, activation, and survival. The anticancer activity of these GD2/IL-15 CAR-T cells was enhanced, and on day 70, 60% of mice were tumor-free. This was remarkable in contrast to the use of unmodified GD2 CAR-T cells where the tumors had persisted in all mice. They also assessed a hydrogel drug delivery system that allowed for prolonged CAR-T-cell distribution as opposed to a single sub-retinal injection. The hydrogel form of the GD2/IL-15 CAR-T cells totally stopped tumor development, improved CAR-T-cell persistence, and revealed decreased structural retinal damage upon histologic analysis^23^.

Although CAR-T-cell therapy carries exciting potential, there are several factors to contemplate when evaluating its potential medical uses. The foremost concern is about immune system adaptability and escape, as CAR-T-cell antigen downregulation by tumor cells has been observed^24^. In the Andersch research, there was a sharp decline in CD171 expression when retinoblastoma cell lines were grown with CD171 CAR-T cells^21^. This downregulation of antigens illustrates the necessity of effectively targeting multiple antigens.

Similarly, in the Sujjitjoon investigation, when more tumor cells were added to imitate a high tumor burden, tumor cells were still proliferating after 6 days, with decreased GD2 expression. Moreover, the CAR-T cells expressed programmed death protein 1 (PD-1), and the tumor cells upregulated programmed death ligand 1 (PD-L1), both of which were absent at baseline, authors believe it decreased the CAR-T cells cytotoxicity and permitted retinoblastoma cells to escape^22^.

### Uveal melanoma

Uveal melanoma (UM) is the most prevalent primary intraocular tumor in adults, originating from the melanocytes in the uveal tract of the eye. Treatment strategies include enucleation, stereotactic radiotherapy, brachytherapy, and proton therapy^25^. However, metastasis to other sites, such as the liver, is inevitable in around half of the patients. While modalities like immunotherapy and BRAF inhibitors are gaining success in the treatment of cutaneous melanoma, the same cannot be said for UM^26^. Persistently poor prognosis of Metastatic UM has led to the exploration of novel therapies like CAR-T cells.

CAR-TIL therapy is a promising approach for treating melanoma patients. It involves engineering tumor-infiltrating lymphocytes (TILs) to express CAR antigens and has shown success in eradicating melanoma in patient-derived xenograft (PDX) mice^27^. To further test the safety, a first-in-dog trial was conducted to treat four companion dogs with autologous anti-HER2 CAR-TILs. The previous mice study showed IL-2 is necessary for therapy effectiveness, hence IL-2 was also administered. The findings indicate that these cells were well-tolerated and demonstrated signs of anti-tumor activity. There were only mild negative effects, all linked to concurrent IL-2 treatment, with symptoms mainly concentrated in the gastrointestinal tract. Lowering the IL-2 dose controlled toxicity, which ceased once the administration was stopped. CAR-TILs were not detected in dog blood, with future studies needed to determine if they reach the target. This innovative treatment strategy expands the tumor-targeting capacity of TILs and offers hope for patients who do not respond to other treatments^28^.

Moreover, The GAIL-N trial is an early phase I study that will recruit 94 patients, including those with metastatic uveal melanoma. The purpose of this trial is to assess the largest safe dose, toxicity, and efficacy of autologous T lymphocytes that express GD2-specific chimeric antigens to treat GD2-positive solid cancers. (NCT03635632)^29^

Application of CAR-T-cell therapy for the treatment of ocular tumors, such as retinoblastoma and uveal melanoma, will likely face numerous issues similar to the ones in solid organ malignancies, which include a limited number of target antigens and heterogeneous antigen expression, a lack of effective T-cell trafficking to tumor sites, and an immunosuppressive tumor microenvironment^30^. While CAR-T-cell therapy carries promising potential, several challenges will need to be met before it can be considered an effective clinical therapeutic strategy.

### Acute lymphoblastic leukemia relapse

Acute lymphoblastic leukemia (ALL) is a hematological malignancy, characterized by the overproduction of immature white blood cells called lymphoblasts. Leukemia frequently involves the eye and orbit, a sign of a bad prognosis^31^. The current treatment strategy comprises chemotherapy, stem cell transplantation, and irradiation. Novel treatments like CAR-T-cell therapy, monoclonal antibodies, and Tyrosine kinase inhibitors (TKI) have emerged as new promising options for the treatment of ALL relapses^32^.

A case report has described a unique case of acute lymphoblastic leukemia relapse that was confined to the anterior chamber of the eye. The novel CAR-T-cell therapy, combined with radiotherapy, was used to treat a 21-year-old male patient who presented with anterior chamber cells and pseudo-hypopyon in his left eye, 4 months after completing chemotherapy and a bone marrow transplant. On the last examination, after 12 months of CAR-T-cell therapy, the patient showed excellent ocular results, with outstanding visual acuity in both eyes and no recurrence of ocular or systemic disease^33^.

The outcome of this combination therapy was remarkable; however, it is more likely that the therapeutic benefits were mainly a result of irradiation rather than CAR-T-cell therapy, as, there are several case reports recounting intraocular tumor relapse after CAR-T-cell therapy, due to lack of T-cell trafficking to the intraocular sites^34–37^. To comprehensively review the safety and efficacy of CAR-T-cell therapy in intraocular relapses of ALL, a clinical trial is necessary.

### Neuromyelitis spectrum disorder (NMOSD)

Recent advancements in the understanding of diseases have expanded the potential for cellular immunotherapies beyond oncology. New genetic engineering technologies allow for revolutionary clinical approaches to previously untreatable diseases, such as neuromyelitis optica spectrum disorder (NMOSD)^4^

NMOSD is an autoimmune disorder causing inflammation and damage to the spinal cord and optic nerves. Optic neuritis is a common symptom leading to severe vision loss if untreated^38^. IgG autoantibodies against Aquaporin 4 water channels play a role in its pathogenesis. Acute attacks are treated with corticosteroids and exchange plasmapheresis, while azathioprine is used to prevent relapses. Recently, a few monoclonal antibodies have been approved, after effectively treating NMOSD. However, some monoclonal antibodies against B-lymphocyte antigen CD20, were unable to lower AQP4-IgG concentrations^39^. Therapies targeting AQP4-IgG-producing cells may be more successful, for AQP4-IgG-seropositive NMOSD, which has led to the investigation of CAR-T-cell therapy.

An open-label phase I clinical study is currently underway, testing BCMA CAR-T-cell treatment for refractory AQP4-IgG-seropositive NMOSD, in twelve patients^40^. (ClinicalTrials.gov NCT04561557). Among the main outcome measures are adverse events and dose-limiting toxicities. The patients showed a safe and easily manageable profile. Mainly cytopenia and infection were experienced as adverse events, but the severity and duration of cytopenia were lower compared to MM patients^41,42^, possibly due to less myelosuppression in NMOSD. Infections were reported but did not exceed the expected severity. Moreover, the initial results of the clinical study show a notably positive clinical response, even without the need for any additional immunosuppressive therapy. 11 patients had no relapse in 5.5 months and reported an overall improvement in disabilities and quality-of-life outcomes. However, the pre-infusion lymphodepletion regimen may also have lowered the relapse rates. Long-term follow-up will indicate whether CAR-T-cell therapy can offer long-lasting disease control for AQP4-IgG-seropositive NMOSD without the need for additional immunosuppressive therapy^40^.

## Ocular adverse events in chimeric antigen receptor T-cell therapy

### General ophthalmic complications of CAR-T-cell therapy

CAR-T-cell therapy has demonstrated promising therapeutic results, but it also carries the risk of unusual toxicities. The most prominent treatment-related hazard is cytokine release syndrome (CRS), a clinical condition caused by CAR-T-induced rapid immune activation. CRS begins with a fever and can escalate to a life-threatening capillary leak with hypoxia and hypotension, potentially resulting in multiorgan failure. The clinical manifestations of CRS are directly linked to the activation of T cells and significant increases in serum inflammatory markers and cytokines. Tocilizumab, an anti-IL-6 receptor antagonist, and steroids are the recognized treatments for CRS, however, the most effective timing for their administration requires further study and clarification^43^.

Neurotoxicity, known as Immune effector cell-associated neurotoxicity syndrome (ICANS), is another frequent adverse event. The potential for neurotoxicity after CAR-T-cell therapy ranges from encephalopathy to seizures, obtundation, and even death. While the pathophysiology of ICANS is not yet fully understood, it is typically observed in tandem with CRS symptoms. Endothelial activation is believed to be a significant factor in neurotoxicity, as heightened permeability of the blood-brain barrier (BBB) allows for the entry of pro-inflammatory cytokines like IFNγ, as well as CAR-T cells themselves, into the central nervous system (CNS)^44^.

### Ophthalmic complications of CAR-T-cell therapy

Our findings, presented in Table 1, includes case report findings to offer a comprehensive understanding of ocular adverse effects associated with CAR-T-cell therapy. Case reports provide detailed descriptions of rare or unusual occurrences, offering valuable insights into potential adverse events that may not be captured in larger studies or clinical trials. They are valuable for identifying new diseases, unusual side effects, or complications of treatments that might not be seen in controlled environments like clinical trials. Since CAR-T-Cell therapy is a relatively novel therapy, case studies were proven to be particularly informative and more prevalent in database searches for the aforementioned reasons

**Table 1:** Ophthalmic complications of CAR-T-cell therapy.

Case report (year)	Age/sex	Past medical history	Adverse presentation	Clinical findings	Investigation and diagnosis
Abou-Samra *et al.* ^35^ (2023)	62/M	Multi treatment resistant DLBCL	Papillitis and vitritis upon completion of CAR-T therapy	IOP 18/17 mm Hg. Fundus revealed 1–2+vitreous cell, disc edema, and mild peripheral vascular sheathing	MRI: multiple new lesions in the bilateral cerebral hemispheres and thalami. Blood workup reactive for CMV and toxoplasmosis. Flow cytometry: kappa monotypic CD5+and CD10- B-cells.
Taher *et al.* ^36^(2023)	59/M	DLBCL	Non-specific RE floaters and discomfort	OCT revealed sub-retinal saw-tooth deposits in the RE	
Case-series Lee *et al.* ^45^ (2021)	Aged 13–78	7 case reports of previously diagnosed Pre BALL/BALL	Decreased VA, NLP and RAPD	Rare blast cells seen. Thickening and enhancement of ON.	LP, CT, MRI of the brain and orbit. Findings consistent with inflammatory response driven by the CAR-T-cell destruction
Huang *et al.* ^46^ (2022)	52/F	Relapsed pre-B ALL	Headache, fluctuating consciousness, blurred vision and pain in LE	NLP and RAPD. Disc swelling and sub-retinal fluid in LE.	Flow cytometry of aqueous humor: T-cell component, favoring CAR-T-cell infiltration
Veys *et al.* ^37^ (2020)	9/M	BALL	Undergoing treatment for adverse effects due to CAR-T-cell therapy including chest infections and agitative signs.	Enhancing lesion in the RE on CT. RE VA reduced.	Vitrectomy with tumor and vitreous biopsy: relapse of CD19+leukemia. 10 mm optic nerve invasion including pia mater
Bin dokhi *et al.* ^47^(2022)	43/F	Primary R-DLBCL (stage III A).	Metamorphopsia and sudden blurring in the vision of the LE.	VA: 20/25 in the LE. Diffuse yellowish retinal infiltration.	CMV quantitative titers and absolute CD4+cell increased. OCT of the LE: Destruction of all retinal layers and active retinitis. Fundus showed creamy infiltrate involving the macula
Zu *et al.* ^48^ (2022)	58/M	MM (stage IIA)	Decreasing VA in RE	VA in RE: 20/800, mutton-fat keratic precipitates, and aqueous flare	Vitreous humor revealed the presence of CMV DNA. CMV retinitis and RD was diagnosed
Alsarhani *et al.* ^49^ (2022)	53/M	R-DLBCL	VZV skin infection associated with blurred vision in the right eye.	Fundus: patches of scattered white retinitis	Skin punch biopsy to confirm the diagnosis of VZV infection.
Willier *et al.* ^34^ (2020)	5 months/M	R-BCP-ALL	Erythema of the LE with pain, hyposphagma, vision impairment, and ptosis within 1 wk	Conjunctival chemosis, AC cellular infiltration and increased IOP	Biopsy of episclera and conjunctiva and AC tap: AC filled with a pus-like yellowish fluid. Episcleral, intraocular and lamina propria infiltration by atypical CD19+cells.
Kochenderfer *et al.* ^50^ (2017)	22 patients treated	DLBCL	Vision loss 3 mo after CAR-T-cell infusion in 1 patient	Clinical course and electroretinography: consistent with fludarabine toxicity	
Denton *et al.* ^51^ (2020)	13/F	BALL	Worsening VA in the LE and NLP in the RE. Later developed significant PSC, after 6 mo.	Total bilateral exudative RD, intraretinal hemorrhage, retinal whitening, and 4+optic disc edema.	CSF showed leukocytosis and local CAR T-cell expansion and cytokine release was postulated
Khanna, *et al.* ^52^ (2022)	Exudative Retinal Detachment Following CAR-T-Cell Therapy in Relapsed B-Cell Acute Lymphoblastic Leukemia				Article not Available

AC, anterior chamber; ALL, acute lymphoblastic leukemia; APD, afferent pupillary defect; CAR, chimera antigen receptor; CMV, cytomegalovirus; CSF, cerebrospinal fluid; CT, computed tomography; DLBL, Diffuse large B-cell lymphoma; DNA, deoxyribonucleic acid; F, female; HBV, hepatitis B virus; HCV, hepatitis C virus; HIV, human-immune virus; IOP, intraocular pressure; LE, left eye; LP, lumbar puncture; M, male; MM, multiple myeloma; NLP, no light perception; OCT, optical coherence tomography; ON, optic nerve; Op, opening pressure; PSC, posterior sub-capsular cataracts; RAPD, relative afferent pupillary defect; R-BCP-ALL, relapsed/refractory B-cell precursor acute lymphoblastic leukemia; RD, retinal detachment; RE, right eye; U/S, ultrasound; VA, visual acuity; VZV, Varicella Zoster Virus.

Several case reports have brought attention to the recurrence of hematological malignancies within the eye following CD19 CAR-T-cell therapy, despite successful control of the central nervous system and the rest of the body^35,36,45^. It is suggested that insufficient anti-CD19 CAR-T cells may enter the eye, allowing neoplastic B cells to persist. Additionally, ocular organs that express FasL and lack MHC1 might be preventing CAR-T cells from entering the eye^53^. This exclusion of CAR-T cells from immune-privileged intraocular locations via FASL could play a role in the development of lymphoma in ocular tissues.

Optic neuropathy post- CAR-T-cell therapy may be linked to ICANS, and a definitive treatment strategy for it has not yet been established^46^. Activation of CAR-T cells can disrupt the blood-brain barrier and blood-ocular barrier, leading to cytokine and T-cell accumulation in the CNS, retina, and optic nerve, resulting in neurotoxicity and ocular complications^53^.

Infection reactivation is also observed post-CD19 CAR-T-cell therapy. Prolonged cytopenia from prior immunomodulatory drug use weakens the immune system, making patients susceptible to infections. A study reported a one-year incidence of bacterial and viral infections at 57.2% and 44.7%, respectively, following CAR-T-cell treatment^54^. Incidences of cytomegalovirus retinitis and varicella zoster virus retinitis have been reported in patients who underwent CAR-T-cell therapy. However, it is reassuring to note that these patients responded positively to ganciclovir treatment^47–49^. It is important to closely monitor patients who undergo CAR-T-cell therapy for potential viral infections and promptly initiate appropriate treatment when necessary.

## Strategies and solutions

As Table 1 demonstrates, the most relevant differential diagnoses in patients who have undergone CD19 CAR-T-cell therapy and present with ocular symptoms are an intraocular relapse of malignancy and intraocular infections. Therefore, it’s important to consider their possibility in such patients, especially if the symptoms progress rapidly, and to initiate appropriate investigations to ensure the correct diagnosis and differentiation between leukemia-related and non-leukemia-related ocular symptoms.

Due to the immune-privileged location of the eye, there is a possibility of intraocular relapse despite effective CAR-T-cell response in the bone marrow and CNS^34,35,55^. Physicians should consider screening with eye examinations before therapy to identify at-risk patients. Routine ophthalmic follow-up after CAR-T-cell therapy can aid in early identification and intervention to prevent deadly relapses. Similar to ICANS, CAR-T cells may damage the blood-ocular barrier, leading to complications like optic neuropathy^45,46^. Understanding the pathophysiology of these ocular complications and developing innovative management techniques is essential to minimize ocular toxicities while maintaining anti-tumor activity and effective T-cell signaling.

The increasing incidence of post-CAR-T infections^47–49^ emphasizes the need for further research on their risk factors, clinical features, diagnosis, prophylactic techniques, and management. As novel CAR-T-cell therapies continue to evolve, the risk of ocular adverse events associated with each new product remains unclear. More data on the risk of ocular side effects and long-term complications is needed to guide preventative strategies, screening protocols, and interventions for these adverse events as this therapy becomes more widely used for different illnesses in the future.

## Conclusion

Despite being effectively introduced decades ago, CAR-T-cell therapy is still evolving. It has proved effective against the treatment of hematological malignancies, and efforts are now being made to combat solid tumors, particularly those of the eye, such as retinoblastoma and melanoma. Although excitement in cell therapy persists even today, much has been overshadowed by the reported neurotoxicity and persistently growing complaints of ophthalmic complications. Thus, further research, extensive monitoring of both pre-therapy and post-therapy infusions, and practical guidelines for this novel therapy must be drafted. Patients receiving this therapy should also be counseled to report any side effects and to seek medical help for early therapeutic intervention.

## Ethical approval

Ethical approval was not applicable for this review

## Consent

Informed consent was not applicable for this type of review.

## Source of funding

No funding was required for this type of review

## Author contribution

All authors equally contributed in this review

## Conflicts of interest disclosure

No conflicts of interests were present in this study

## Research registration unique identifying number (UIN)

Human participants were not involve in this review, and hence do not require any registration.

## Guarantor

Sara Sarwar.

## Data availability statement

Not applicable.

## Provenance and peer review

Not commissioned, externally peer-reviewed
